# A Novel Approach of Harvesting Viable Single Cells from Donor Corneal Endothelium for Cell-Injection Therapy

**DOI:** 10.3390/cells9061428

**Published:** 2020-06-09

**Authors:** Hon Shing Ong, Gary Peh, Dawn Jin Hui Neo, Heng-Pei Ang, Khadijah Adnan, Chan Lwin Nyein, Fernando Morales-Wong, Maninder Bhogal, Viridiana Kocaba, Jodhbir S. Mehta

**Affiliations:** 1 Tissue Engineering and Cell Therapy Group, Singapore Eye Research Institute, Singapore 169856, Singapore; gary.peh.s.l@seri.com.sg (G.P.); dawn.neo.j.h@seri.com.sg (D.J.H.N.); ang.heng.pei@seri.com.sg (H.-P.A.); khadijah.adnan@seri.com.sg (K.A.); kaekae.sg@gmail.com (C.L.N.); fernando.morales.wong@snec.com.sg (F.M.-W.); manibhogal@aol.com (M.B.); kocaba@niios.com (V.K.); 2 Eye-Academic Clinical Program (ACP), Duke-National University of Singapore (NUS), Graduate Medical School, Singapore 169857, Singapore; 3 Corneal and External Diseases Department, Singapore National Eye Centre, Singapore 168751, Singapore; 4 Cornea Unit, Guy’s & St Thomas’ Hospital, London SE1 7EH, UK; 5 Netherlands Institute for Innovative Ocular Surgery, Melles Cornea Clinic, Amnitrans EyeBank Rotterdam, 3071 AA Rotterdam, The Netherlands; 6 School of Material Science and Engineering, Nanyang Technological University, Singapore 639798, Singapore

**Keywords:** ophthalmology, cornea, cell injection, cell therapy, corneal endothelium, regenerative medicine, corneal edema, bullous keratopathy, corneal transplantation, keratoplasty, eye banking, tissue engineering

## Abstract

Donor corneas with low endothelial cell densities (ECD) are deemed unsuitable for corneal endothelial transplantation. This study evaluated a two-step incubation and dissociation harvesting approach to isolate single corneal endothelial cells (CECs) from donor corneas for corneal endothelial cell-injection (CE-CI) therapy. To isolate CECs directly from donor corneas, optimization studies were performed where donor Descemet’s membrane/corneal endothelium (DM/CE) were peeled and incubated in either M4-F99 or M5-Endo media before enzymatic digestion. Morphometric analyses were performed on the isolated single cells. The functional capacities of these cells, isolated using the optimized simple non-cultured endothelial cells (SNEC) harvesting technique, for CE-CI therapy were investigated using a rabbit bullous keratopathy model. The two control groups were the positive controls, where rabbits received cultured CECs, and the negative controls, where rabbits received no CECs. Whilst it took longer for CECs to dislodge as single cells following donor DM/CE incubation in M5-Endo medium, CECs harvested were morphologically more homogenous and smaller compared to CECs obtained from DM/CE incubated in M4-F99 medium (*p* < 0.05). M5-Endo medium was hence selected as the DM/CE incubation medium prior to enzymatic digestion to harvest CECs for the in vivo cell-injection studies. Following SNEC injection, mean central corneal thickness (CCT) of rabbits increased to 802.9 ± 147.8 μm on day 1, gradually thinned, and remained clear with a CCT of 385.5 ± 38.6 μm at week 3. Recovery of corneas was comparable to rabbits receiving cultured CE-CI (*p* = 0.40, *p* = 0.17, and *p* = 0.08 at weeks 1, 2, and 3, respectively). Corneas that did not receive any cells remained significantly thicker compared to both SNEC injection and cultured CE-CI groups (*p* < 0.05). This study concluded that direct harvesting of single CECs from donor corneas for SNEC injection allows the utilization of donor corneas unsuitable for conventional endothelial transplantation.

## 1. Introduction

Corneal diseases are a leading cause of blindness [1,2]. A significant proportion of corneal blindness is the result of a dysfunctional corneal endothelium (CE), of which Fuchs endothelial corneal dystrophy (FECD) and pseudophakic bullous keratopathy (BK) are the two commonest causes [2,3]. The human CE, the innermost cellular monolayer of the cornea, plays a crucial role in the dynamic maintenance of corneal hydration, keeping the cornea transparent through a leaky barrier of tight junctions and active ionic pumps [4,5,6,7,8]. As human CECs are known to have limited capacity to regenerate in vivo [9], any significant loss of CECs due to diseases or iatrogenic damage will result in the spreading and enlargement of the remaining CECs to maintain functional integrity of the CE [10,11]. However, when endothelial cell density (ECD) falls below a critical threshold, detrimental enough to reduce the functional capacity of the CE, the cornea becomes edematous, affecting corneal transparency. This eventually leads to corneal blindness [10,11].

Visual impairment from corneal endothelial dysfunction is one of the most common indications for corneal transplantation worldwide [3,12,13]. Modern techniques of endothelial transplantation, namely Descemet’s stripping automated endothelial keratoplasty (DSAEK) and Descemet’s membrane endothelial keratoplasty (DMEK), can achieve high surgical success rates. Compared to traditional full-thickness penetrating keratoplasty (PK), endothelial keratoplasty (EK) techniques are reported to have better visual outcomes, lower risks of graft rejection, and superior graft survival outcomes [14,15,16,17,18,19]. Unlike PK, DSAEK/DMEK techniques use smaller incisions, avoiding full-thickness corneal trephination and intraoperative “open-sky” situations which are associated with risks of sight-threatening complications such as expulsive hemorrhage [20]. Being minimally invasive, DSAEK/DMEK also preserves the biomechanical integrity of the cornea. In the event of trauma, the risks of sight-threatening open-globe injuries are higher in eyes that have undergone PK compared to eyes that have undergone DSAEK/DMEK, as inherent weaknesses exist at the graft–host junctions of PK eyes. Thus, DSAEK and DMEK are now considered the standard of care for the treatment of corneal blindness from endothelial diseases.

However, the number of corneal transplants performed annually is significantly limited by a global shortage of donor corneas suitable for transplantation [21]. A survey published in 2016 revealed that, although 184,576 corneal transplants were performed between 2012–2013, an estimated 12.7 million individuals were still waiting for sight-restoring transplant surgeries [2]. This implied that only 1 in 70 of the overall needs of corneal transplantation were met [2]. Within the same report, the authors revealed that, out of the 283,530 corneas procured in 82 countries, approximately 35% of the corneas were not used due to either (1) low ECD or (2) positive infectious serological testing. Furthermore, it was also estimated that over 50% of patients worldwide have no access to corneal transplantation [2]. As ECD and donor-tissue quality is inversely related to donor age, some surgeons are reluctant to use corneal tissue retrieved from older donors for conventional corneal transplantation. To avoid tissue wastage and the unnecessary cost of retrieval, many eye banks have set upper age-limits for donor eligibility [22,23]. With an overall increasing life expectancy across nations worldwide [24], it is inevitable that the average age of donors will rise [25]. As such, the numbers of donor corneas harvested that are usable for transplantation will most likely decline [25,26,27].

With these restrictions in donor-reliant transplantations, there is a strong drive to search for alternative treatment modalities, such as scalable cell-based therapies, to meet the global demands for corneal transplantation [28,29,30,31,32,33,34]. Current research in corneal cell-based therapies involves the delivery of functional human CECs isolated from younger donor corneas and propagated in culture [31,32,33,34,35,36,37,38]. Currently, there are two main approaches to deliver such propagated cells: either via (1) cell injection or (2) as a tissue-engineered construct [31,32,33,34,35,36,37,38]. In the cell-injection approach, cultured human CECs are delivered by direct injection of the cells into the anterior chamber. This is followed by at least three hours of posturing facedown to allow the injected CECs to adhere onto the posterior corneal surface [31,32,33,34,35,36,37,38,39]. Investigators have also described the use of magnetic attachment of iron-endocytosed CECs [33], superparamagnetic microspheres [40], or magnetic nanoparticles [41] to aid in the distribution and adherence of injected cells. The tissue-engineered construct approach involves engineering the cells onto a suitable scaffold carrier; this is then transplanted into the eye, using techniques that are similar to current EK techniques [36,37,42].

In 2018, a landmark study reporting the clinical efficacy of cell-injection therapy for the treatment of bullous keratopathy in humans was published [38,43]. Investigators found that the intracameral injection of cultured human CECs suspended in growth medium supplemented with rho-associated protein kinase (ROCK) inhibitor (Y-27632) successfully reversed corneal edema, with clinical outcomes being stable up to two years postoperatively [38]. In our recent publication, we demonstrated the functionality of a single technique of expanded CECs, delivered by either cell-injection or tissue-engineered approaches, within a rabbit model of BK [37,42]. Institutional review board (IRB)-approved clinical trials investigating cell-based therapies for corneal endothelial diseases are currently underway (www.clinicaltrials.gov NCT04319848). Nevertheless, obtaining human CECs expanded in culture requires specialized laboratory facilities and trained personnel with the appropriate expertise, specifically the ability to propagate CECs within an accredited Good Manufacturing Practices (GMP) environment [21,28,29,30,42]. Regulatory safety standards must also be met in order to use these cultured CECs in human clinical trials [42].

An alternative to the expansion of human CECs in culture and another means of maximizing donor corneal tissue availability may be the repurposing and utilization of donor corneas deemed unsuitable for endothelial transplantation. For endothelial transplantations of DSAEK and DMEK, given the inevitable iatrogenic cell loss of donor graft observed in the perioperative transplantation period [19], donor corneas must have a minimum threshold of ECD to ensure that the remaining transplanted cells are able to adequately function to maintain corneal transparency over time following transplantation [44,45]. As such, donor corneas with low ECD (<2500 cells/mm^2^) are generally deemed unsuitable for EK procedures [44,45]. Furthermore, there is also a pool of donor corneas that are often excluded by eye banks for use in specific EK procedures such as DMEK. DMEK is the most recent advancement in EK procedures where only donor Descemet’s membrane (DM) and CE are harvested and transplanted into the recipient [46]. Certain donor characteristics such as young donor age [47,48,49], diabetes mellitus [50,51], or old DM scars [52] can affect DM adherence and fragility, increasing the difficulties of harvesting an intact undamaged DMEK graft. Thus, despite having sufficiently high ECD, these donor corneas are often rejected for DMEK surgery.

We hypothesized that viable and functional CECs for cell-injection therapy could be harvested from donor corneas that are unsuitable for conventional corneal endothelial transplantations. In this preclinical study, we introduced a simple approach of harvesting CECs from donor corneas through a two-step incubation and dissociation technique. Unlike published methodologies, this approach does not require complex cellular propagation techniques. It involves the isolation of the DM, incubation of the peeled DM/CE within a stabilization medium for at least 48 h, and dissociation of CECs into single cells through mild enzymatic digestion. Using our established rabbit model of BK, we then showed that these dissociated CECs could be pooled into a concentration sufficient for a simple non-cultured endothelial cell (SNEC)-injection therapy for the treatment of corneal endothelial dysfunction [37,42].

## 2. Materials and Methods

### 2.1. Study Design

This study was approved by the local centralized institutional review board (SingHealth IRB reference: 2016/2839). There are two main objectives to this study. The first objective was to characterize and establish the two-step incubation and dissociation approach of isolating pure populations of single CECs from research grade donor corneas through a series of donor-matched comparisons. Morphometric analyses of cultured cells following isolation were assessed. The second objective was to demonstrate the functionality of the isolated single cells using a rabbit model of BK through a cell-injection approach as previously described [37]. New Zealand white rabbits were used, and their care and treatment strictly adhered to the regulations of The Association for Research in Vision and Ophthalmology (ARVO) statement for Use of Animals in Ophthalmic and Vision Research. All experimental procedures were approved by the local SingHealth Institutional Animal Care and Use Committee (IACUC reference: 2017/SHS/1343). Following the removal of the host rabbit CE via scrapping, the functionality of the injected single cells was assessed for their capacity to rescue the insult and to keep the cornea clear. Rabbits receiving the harvested non-cultured single cells were known as rabbits that received “SNEC injection”. There were two control groups: a positive control group, where the BK rabbits received an injection of CECs propagated in culture (regular “CE-CI”), and a negative control group, where the BK rabbits received an injection of solution with no CECs (“no cells” control). The positive control would allow the evaluation of the efficiency of SNEC injection of CECs compared to regular CE-CI from cultured CECs; the negative control would indicate any occurrence of or dictate the rate of spontaneous recovery as a result of the rabbits’ host CE [53]. Slit lamp images were obtained and corneal thicknesses were measured throughout the study. At the week-3 end point, corneas of rabbits were processed and assessed for the expression of human specific nuclei by immunohistochemistry.

### 2.2. Materials

Ham’s F12, Medium 199, Human Endothelial-SFM, Dulbecco’s Phosphate-Buffered Saline (PBS), TrypLE^TM^ Select (TS), gentamicin, amphotericin B, penicillin, and streptomycin were purchased from Life Technologies (Carlsbad, CA, USA). Insulin/Transferrin/Selenium (ITS) was purchased from Corning (Corning, NY, USA), and ascorbic acid was purchased from Avantor (Radnor Township, PA, USA). Collagen IV from human placenta and Trypan blue (0.4%) were purchased from Sigma (St. Louis, MO, USA). Recombinant human basic fibroblast growth factor (bFGF) and rho-associated, coiled-coil protein kinase inhibitor Y-27632 were purchased from Miltenyi Biotec (Bergisch Gladbach, Germany). FNC coating mixture was obtained from United States Biologicals (Swampscott, MA, USA). Liberase TH was purchased from Roche (Mannhein, Germany). EquaFetal®, the bovine serum used to supplement the culture medium, was from Atlas Biologicals (Fort Collins, CO, USA).

### 2.3. Research-Grade Human CorneoScleral Tissues

All research-grade human cadaver corneal tissues were procured from either Lions Eye Institute for Transplant and Research (Tampa, FL, USA) or Miracles in Sights (Winston-Salem, NC, USA), with informed consent from the next of kin. All research performed with human-derived tissue was carried out in accordance to the principles outlined in the Declaration of Helsinki. All corneoscleral donor tissues were preserved and transported in Optisol-GS (Bausch & Lomb, Rochester, NY, USA) at 4 °C until they were processed.

### 2.4. Corneal Endothelial Cell Culture for Regular CE-CI 

For regular “CE-CI”, primary CECs were isolated from pairs of donor corneas propagated using a dual media approach to the second passage as previously described [30,42]. The CECs were dissociated and resuspended at a concentration of 6.0 × 10^5^ cells in 150 µL of M5-Endo medium containing ROCK inhibitor Y-27637 within a 1 mL syringe attached to a 30-gauge needle in preparation for cell injection [37]. This suspension of CECs containing ROCK inhibitor for cell injection therapies to improve cellular adhesion and to prevent apoptosis has been well-established [37,38,39]. All cultures were maintained within a humidified atmosphere at 37 °C and 5% CO_2_.

### 2.5. Preparation of Single-Cell Human CECs for Characterization or Cell-Injection Surgery

The DM/CE from paired donor corneas were carefully peeled under a stereoscopic dissecting microscope (Nikon, Tokyo, Japan) as previously described [28]. Following isolation of the DM/CE pieces, they were collected and incubated in a serum-supplemented media for at least 48 h.

For initial donor-matched comparison, DM/CE pieces isolated from one donor cornea were incubated in M4-F99 medium (Ham’s F12/M199, 5% serum, 20 μg/mL ascorbic acid, 1 × ITS, and 10 ng/mL bFGF), whereas the isolated DM/CE pieces from the contralateral donor-matched cornea were incubated in M5-Endo medium (Human Endothelial-SFM supplemented with 5% serum). Following incubation, each condition media was replaced with TS (1×) and incubated for between 30 to 50 min, with periodic checks for the status of trypsinization based on the cellular morphologies of CECs—every 10 min after the first 20 min, as the rate of trypsinization varied between donor corneas. Upon dislodgement of the majority of CECs on the DM, the DM/CE pieces were lightly agitated with a 1-mL pipette before a final incubation of 5 min. Thereafter, the respective media comprising of the trypsinized cells were collected and passed through a pre-wet 100-µm filter, before being resuspended in an Eppendorf tube. For characterization studies, filtered cells were then seeded within an FNC-coated 12-mm glass area of a Willco culture dish and incubated in M5-Endo medium for 4 weeks, where images were taken for morphometric analysis as previously described [29]. Briefly, cellular morphologies of CECs were captured using a Nikon TS1000 phase contrast microscope with a Nikon DS-Fil digital camera (Nikon, Minato City, Tokyo, Japan). Morphometric data of the area and perimeter of randomly selected cells from phase contrast images were manually outlined by point-to-point tracing of the cell borders using ImageJ software [54]. Cell circularity was then determined using the formula: circularity = (4π × area)/perimeter^2^, where a value approaching 1.0 indicates a circular profile. Hence, hexagonal CECs will have a profile closer to 1.0 compared to CECs of irregular shapes.

For subsequent cell-injection studies, it should be noted that all incubations of isolated DM/CE pieces from pairs of corneas from a single donor were pooled and incubated in M5-Endo medium for at least 48 h. Given the theoretical risk of rejection with multiple allogenic antigen exposure, we did not pool cells obtained from different donors. Following trypsinization and passing through the 100-µm filter, single cells were resuspended in an Eppendorf tube before they were prepared in a volume of 150 µL of M5-Endo containing 10 µM of a Rho-associated kinase inhibitor, Y-27632.

### 2.6. Animal Surgeries

New Zealand white rabbits (*n* = 12) used in this study were separated into a treatment group of rabbits receiving SNEC injection (*n* = 4), a positive control group of rabbits receiving regular cultured CE-CI (*n* = 4), and a negative control group of rabbits receiving an injection of solution containing Y-27632 without CECs (*n* = 4). Lens extraction surgeries were performed by H.S.O. and F.M.-W., and cell-injection procedures were performed by J.S.M., V.K., and H.S.O. All surgical procedures and follow-up evaluations were performed under general anesthesia achieved by intramuscular injections of 5 mg/kg xylazine hydrochloride (Troy Laboratories, New South Wales, Australia) and 50 mg/kg ketamine hydrochloride (Parnell Laboratories, New South Wales, Australia), along with topical application of lignocaine hydrochloride 1% (Pfizer Laboratories, New York, NY, USA).

### 2.7. Lens Extraction Surgeries

The crystalline lenses of rabbits were extracted by means of a standard phacoemulsification technique using the White Star phacoemulsification system (Abbott Medical Optics, Santa Ana, CA, USA) [37]. Surgeries were performed through 2.8-mm clear corneal incisions. To achieve mydriasis, tropicamide 1% (Alcon Laboratories, Geneva, Switzerland) and phenylephrine hydrochloride 2.5% (Alcon Laboratories, Geneva, Switzerland) eye drops were administered approximately 30 min before lens extraction surgery. Corneal incisions were closed with 10/0 nylon sutures, and the rabbits were left aphakic with an intact posterior capsule for at least one week before the experimental cell-injection procedures.

### 2.8. Simple Non-Cultivated Endothelial Cell (SNEC) and Corneal Endothelial Cell Injection (CE-CI)

The method of delivery of human CECs was based on our previous studies [37]. Briefly, prior to cell injection, a single intravenous dose of heparin (500 units in 1.0 mL; Hospira, Melbourne, Australia) was administered to the rabbits to reduce intraocular fibrin formation. Subsequently, an AC maintainer was placed to infuse a balanced salt solution (BSS) containing additional heparin (1 unit per mL). A paracentesis was then created with a diamond knife to accommodate the insertion of a 30-gauge silicone soft tipped cannula (catalogue number: SP-125053, ASICO, Westmont, IL, USA) for the scrapping of rabbits’ CECs. The aim was complete removal of all rabbits’ CECs from limbus to limbus whilst keeping the DM intact. This was performed for both rabbits in the experimental group and control group. Continuous irrigation with BSS ensured that the endothelial cells did not remain on the surface of the DM. A solution of trypan blue (Vision Blue, Dorc, Zuidland, The Netherlands) was injected intracamerally to aid in the assessment of the DM denudation. Areas of DM devoid of CEs were stained blue, and any areas with residual CE stood out against blue-stained DM. The scraping process was then repeated to target these areas until the entire DM was stained blue, indicating that all corneal endothelial cells had been removed. Subsequently, 0.5 mL of 100 μg/mL carbochol (Miostat^®^, Alcon Laboratories, Geneva, Switzerland) was injected to achieve intraoperative miosis. Both the paracentesis incision and the AC maintainer paracentesis sites were secured with 10/0 nylon interrupted sutures. This was followed by a 0.2 mL anti-inflammatory and anti-infective subconjunctival injection of a 1:1 mixture of 4 mg/mL dexamethasone sodium phosphate (Hospira, Melbourne, Australia) and 40 mg/mL gentamicin sulfate (Shin Poong Pharmaceutical, Seoul, Korea). Using a syringe and 30-gauge cannula, 0.4 mL of aqueous humor was removed to shallow the anterior chamber. CECs suspended in ROCK inhibitor Y-27632 and M5-Endo medium were then injected through a separate tunneled track via a 30-gauge needle. Immediately following cell injection, rabbits were placed in a manner that ensured that the cornea was in a downward position maintained for three hours under volatile anesthesia.

### 2.9. Postoperative Care

Following cell-injection procedures, all rabbits received a postoperative regime of topical prednisolone acetate 1% (Allergan Inc, Dublin, Ireland) and topical antibiotic moxifloxacin hydrochloride 0.5% (Vigamox, Alcon Laboratories, Geneva, Switzerland) four times a day. An intramuscular injection of 1 mL/kg dexamethasone sodium phosphate (Norbrook Laboratories, Newry, Northern Ireland, United Kingdom) was also administered once daily. This medication regime was maintained until the rabbits were sacrificed.

### 2.10. Corneal Imaging and Intraocular Pressure Measurement

All corneal imaging and measurements of intraocular pressures (IOP) were performed prior to transplantation as well as at the following postoperative time-points: day 1, day 4, week 1, week 2, and week 3. Slit lamp photographs were taken with a Zoom Slit Lamp NS-2D (Righton, Tokyo, Japan) and corneal cross-sectional scans, and measurements of corneal thickness were performed using an anterior segment-optical coherence tomography (AS-OCT) system (Optovue, Fremont, CA, USA). Three measurements were taken for the assessment of central corneal thickness (CCT): at the corneal center (0.0 mm) and at 1 mm either side of the center (+1.0 mm, and −1.0 mm); mean values were calculated. Measurements of IOP were performed using a calibrated tonometer (Tono-pen Avia Vet, Reichert Ophthalmic Instruments, Depew, NY, USA). In vivo confocal images were obtained using the Heidelberg Retina Tomography (HRT) 3 system combined with the Rostock Corneal Module (HRT3/RCM; Heidelberg Engineering, Heidelberg, Germany) to evaluate corneal ECD following cell injection, where random areas of between 50 to 100 cells were assessed for cell density using the software within. A minimum of at least three confocal images were evaluated to obtain the corneal ECD.

### 2.11. Analysis of Corneas

All rabbits were followed for 21 days following surgery before being sacrificed under anesthesia with an overdose of 85 mg/kg sodium pentobarbitone (Jurox, New South Wales, Australia) intracardiac injection.

### 2.12. Immunohistochemistry

For immunohistochemistry, excised corneal samples were embedded in frozen section compounds (Surgipath; Leica Microsystems, Nussloch, Germany), and stored at −80 °C until sectioning. Serial sections of 8-µm sections were cut using a HM525 NX cryostat (Thermo Scientific, Waltham, MA, USA) and collected on polylysine-coated glass slides (Thermo Scientific, Waltham, MA, USA). Samples were rinsed and blocked in 5% normal goat serum in PBS for 30 min at room temperature (RT). Subsequently, samples were incubated with the primary antibodies at RT for 2 h or at 4 °C overnight. The primary antibody used was antihuman nuclei antibodies (1:50; Merck Millipore, MA, USA). Samples were then labelled with an AlexaFluor 488 conjugated goat anti-mouse IgG secondary antibody (2.5 µg/mL, Life Technology, Waltham, MA, USA), mounted in Vectashield containing DAPI (Vector Laboratories, Burlingame, CA, USA), and visualized using a Zeiss Axioplan 2 fluorescence microscope (Carl Zeiss, Oberkochen, Germany). At least 6 sections per eye were analyzed.

### 2.13. Statistical Analysis

Data was managed in Excel (Microsoft) and analyzed using Statistical Program for Social Sciences (SPSS^©^) Version 22 (IBM, Armonk, NY, USA). Differences in the distribution of continuous variables between groups were analyzed using the two-tailed independent t-test. When distributions of more than two groups were compared, one-way analysis of variance (ANOVA) with Bonferroni correction was used. Significance level was set at *p* < 0.05.

## 3. Results

### 3.1. Information on Donor Characteristics

The characteristics of research-grade human donor corneas utilized for this study are listed in Table 1.

A total of 22 donor corneas were procured for this study. Serial numbers 1 to 6 were single donor corneas (denoted with an “*”), whereas 7 to 22 were paired donor corneas. Donor age ranged from 4 years old to 71 years old with a median age of 53 years. Days taken from death of donor to the initiation of incubation for harvesting or cell expansion ranged from 5 days to 21 days, with a median of 12 days. Under the column donor utilization, optimization studies were represented by Opt, where the donor corneas were utilized for (A) initial dissociation studies, (B) morphometric analyses, and (C) cell count estimation experiments following isolation. “SNECi” represents donors where corneal endothelial cells were harvested using our non-cultured technique and used for cell-injection therapy for in vivo functionality rabbit studies; “CE-CI” represents donors where cells were cultured for at least 1 passage before cell injection for functionality rabbit studies.

### 3.2. Characterization of Harvested CECs

#### Isolation of Single Cells from Peeled Donor Descemet’s Membrane

**Figure 1:**
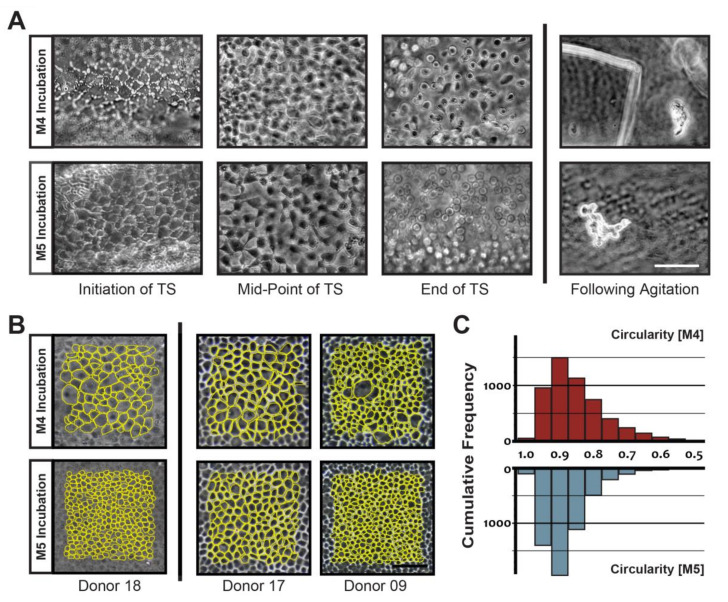
Single cell isolation and morphometric analyses of isolated primary human corneal endothelial cells (CECs): (**A**) Representative images of isolated primary human Descemet’s membrane/corneal endothelium (DM/CE) at various timepoints of dissociation in TrypLE Select (TS), following incubation in either M4-F99 (M4) or M5-Endo (M5) medium for 48 h; (**B**) representative images of confluent donor-matched primary human CECs at four weeks following the initial 48-h incubation in either M4 or M5; the CECs were annotated in Image J (yellow), where manual morphometric analyses were applied; at least 1500 cells were evaluated from each donor (*n* = 3); (**C**) graph showing pooled (*n* = 3) cellular circularity of the donor-matched primary human CECs cultured to four weeks following the two incubation conditions: M4 (represented by red bars) and M5 (represented by blue bars).

Initial attempts to dissociate donor CE as single cells off peeled DM using both enzymatic digestion (collagenase, dispase, papain, ethylenediaminetetraacetic acid (EDTA), and TS) and a combination of chelating agent with manual titrating [55,56] resulted in variable outcomes. The following examples were representative of outcomes after treatment for at least 1 h. Treatment with collagenase resulted in the detachment of the corneal endothelial cells as a whole sheet of cells or larger clusters, whilst treatment with either papain or dispase resulted in the formation of corneal endothelial cell-clusters of various sizes (Supplementary Figure S1; top panel). The use of EDTA by itself was not effective in the detachment of cells, and manual titrating with a flame polished pipette resulted in cells with apparent poor viability and minimal cell attachment (Supplementary Figure S1; bottom panel). As previously reported [28], direct treatment with TrypLE reagent alone or in combination with papain was insufficient for single cell isolation where only partial detachment of single cells was observed in the combination treatment (Supplementary Figure S1; bottom panel). The incubation of the peeled DM/CE in either M4-F99 or M5-Endo media for 48 h prior to TS exposure enabled the successful isolation of single cells with robust consistencies (Figure 1A). When exposed to TS, CECs from peeled DM/CE incubated in M4-F99 medium took a shorter time to dislodge as single cells from the DM (*n* = 3; 38 ± 3 min) compared to CECs from donor-matched tissue incubated in M5-Endo medium (*n* = 3; 53 ± 15 min) (Table 2; Figure 1A). However, following 4 weeks of culture, we observed that single CECs, isolated from DM/CE incubated in M4-F99, were morphologically larger and less homogeneous (mean cell size 1671.7 ± 837.3 µm^2^, coefficient of variance, CV 0.501) compared to donor-matched DM/CE incubated in M5-Endo (mean cell size 998.3 ± 449.3 µm^2^, CV 0.450) (*p* < 0.05) (Table 2; Figure 1B). CECs harvested from DM/CE incubated in M5-Endo were also more circular (circularity index 0.86 ± 0.003 vs 0.83 ± 0.03, *p* = 0.08) (Table 2; Figure 1C). The overall cellular yield was found to be higher in 2 of the 3 donor-matched samples when incubated in M5-Endo than in M4-F99 media (1.83× and 0.27×); there was no difference in the third donor-matched sample (0.03×) (Table 2). As the cellular morphology of established dissociated CECs following incubation in M5-Endo medium were consistently more homogeneous and smaller compared to cultures established following incubation in M4-F99 medium, all subsequent characterization and in vivo functionality studies were performed with 48 h of incubation in M5-Endo medium prior to TS treatment.

To obtain a better estimate of CECs that could be harvested per donor cornea, we isolated DM/CE from an additional 3 donors (a pair of corneas from 1 donor and a single cornea each from 2 different donors) and incubated them separately in M5-Endo medium for 48 h before dissociation using TS treatment. Following cell-straining, manual cell counts indicated a final cell yield of 65,500 cells, 49,168 cells, and 32,500 cells from donor corneas with average ECDs of 2619 cells/mm^2^, 2576 cells/mm^2^, and 1454 cells/mm^2^ respectively. This equated to an arbitrary estimated yield of between 16.7% to 22.1% per donor cornea, factoring in a 25% attrition from procedural cell loss and cell death (Table 3).

### 3.3. Preoperative Assessment of Rabbits Following Cataract Extraction

All rabbits were assessed one week following lens extraction surgery before the planned experimental cell-injection procedures. The corneas of all rabbits were clear with no visible epithelial defects, opacities, or vascularization. No intraocular inflammation was observed.

**Figure 2:**
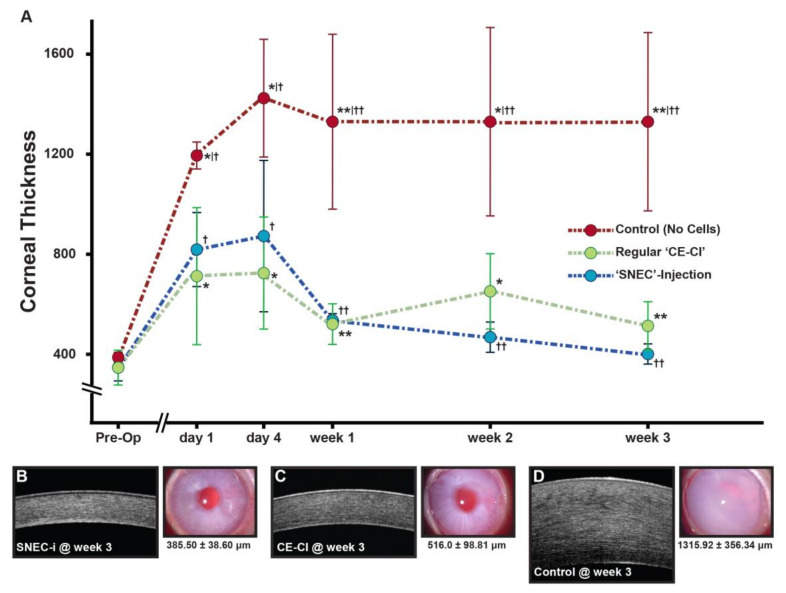
Functional assessment of simple non-cultured endothelial cells (SNEC) injection within a rabbit model of bullous keratopathy: (**A**) Graph summarizing the mean central corneal thickness (CCT) of rabbits that received SNEC injection of CECs (n = 4), regular cultured corneal endothelial cell injection (CE-CI) (n = 4), and “no cells” controls (n = 4) throughout the study period (^*/†^ *p* < 0.05, ^**/††^ *p* < 0.01); representative in vivo anterior segment optical coherence tomography (Optovue, California, USA) and corresponding slit lamp corneal images of (**B**) rabbits that received SNEC injection (SNEC-i), (**C**) rabbits that received regular CE-CI, and (**D**) control rabbits where no cells were injected, all at week 3.

The mean CCTs of rabbits in the “SNEC-injection” treatment group, the regular “CE-CI” positive control group, and “no cells” negative control groups were 331.2 ± 50.5μm (*n* = 4), 352.4 ± 69.0 μm (*n* = 4), and 366.8 ± 18.9 μm (*n* = 4), respectively. There were no significant differences in preoperative CCT among the three groups (*p* = 0.62) (Figure 2A).

### 3.4. Postoperative Clinical Outcomes in Rabbit Model of Bullous Keratopathy

#### 3.4.1. Corneal Transparency

Following the SNEC-injection procedure, there were signs of intraocular inflammation with mild flare observed in the anterior chamber. These resolved within one week after surgery. The corneas of rabbits receiving SNEC injection (Figure 2B) and regular CE-CI (Figure 2C) were progressively clearer throughout the follow-up period, and corneal clarity from both groups of rabbits were maintained throughout the length of the study. The corneas of rabbits in the “no cells” negative control group remained hazy throughout the postoperative period (Figure 2D). No significant IOP elevation was noted in any of the postoperative eyes.

#### 3.4.2. Central Corneal Thickness

Following the SNEC-injection procedure, the mean CCTs of rabbits in this group increased to 802.9 μm ± 147.8 μm on day 1 but were maintained at approximately 853.1 μm ± 305.1 μm on day 4. Subsequent mean CCTs of rabbits in this SNEC-injection group reduced significantly. They were 521.3 μm ± 33.1 μm at week 1, 457.1 μm ± 57.3 μm at week 2, and 385.5 μm ± 38.6 μm at week 3 (Figure 2A). In the CE-CI positive control group, there was an initial increase in mean CCT of rabbits to 711.7 μm ± 273.7 μm on day 1 and to 727.3 μm ± 226.6 μm on day 4, before reducing to 523.3 μm ± 82.3 μm, 653.8 μm ± 148.1 μm, and 516.0 μm ± 98.8 μm at week 1, week 2, and week 3, respectively (Figure 2A). Throughout the experimental period, there were no statistical differences between the mean CCT of rabbits in the SNEC-injection treatment group compared to the regular CE-CI positive control group (*p* = 0.40, *p* = 0.17, and *p* = 0.08 at weeks 1, 2, and 3. respectively). In contrast, the CCTs of rabbits in the “no cells” negative control group were significantly higher (>1000 µm) compared to the other two treatment groups from day 1 (*p* < 0.05), day 4 (*p* < 0.05), and the rest of the follow-up period (*p* < 0.01) (Figure 2A).

### 3.5. Characterization of Excised Corneas: In Vivo Confocal Microscopy and Immunohistochemistry

**Figure 3:**
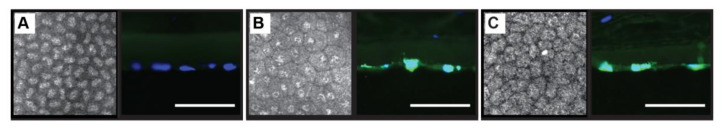
In vivo confocal microscopy and immunohistochemistry of rabbits’ corneas: The in vivo confocal images of corneal endothelium of (**A**) a control rabbit with its own native corneal endothelium, (**B**) a rabbit that received simple non-cultured endothelial cells (SNEC) injection, (**C**) rabbits that received regular cultured corneal endothelial cell injection (CE-CI); all images were taken on the last day of experiment prior to sacrifice. Immunostainings with human-specific nuclei antibodies were performed on the excised rabbits’ corneas and presented alongside the confocal images. Scale bars: 50 μm.

Corneas of rabbits receiving SNEC injections remained significantly thinner throughout the course of the study. Periodic capture of in vivo confocal images revealed a confluent layer of polygonal CECs in a mosaic pattern from postoperative week 1 onwards, which was maintained throughout the follow-up period. Imaged at week 3, it was evident that the rabbits’ CE (Figure 3A), taken from the rabbits’ contralateral eyes, looked vastly different to the monolayer formed by the human CECs that adhered on the posterior corneas of the rabbits receiving SNEC injections (Figure 3B) and regular cultured CE-CI (Figure 3C). Although SNEC injections were able to form functional CE, the monolayers that were established towards the center of the cornea were not compact, with irregularly shaped polygonal cells and a mean central ECD assessed at week 3 of approximately 953 ± 191 cells/mm^2^; in comparison, the mean central ECD of rabbits’ native CE was 2940 ± 29 cells/mm^2^. The mean central ECD of rabbits that received regular CE-CI was 1208 ± 270 cells/mm^2^. These results were confirmed by the immunohistochemistry staining using a human-specific nuclei antibody. No staining was detected in the excised corneas comprising only rabbit CE (Figure 3A), whilst nuclei of the CE from the rabbit that received SNEC injections (Figure 3B) and regular CE-CI (Figure 3C) were positive. It should be noted that the peripheries of the excised corneas from both cell injections were relatively sparse of cells.

## 4. Discussion

Corneal endothelial failure is one of the most common indications for corneal transplantation in the world [3,12,13]. Although the current standard of care for the treatment of corneal endothelial failure through conventional EK surgeries are effective in reversing corneal blindness [1,3,12,13,57,58], the number of transplant surgeries that can be performed is greatly limited by the availability of suitable donor corneas [2]. There is thus a need to search for alternative approaches to treat corneal endothelial failure that are less donor reliant. In this study, we described a simple non-cultivated endothelial cell (SNEC) harvesting approach to obtain single-cell populations of viable CECs directly from donor corneas. The donors used in this study were “research-grade” corneas, some of which had ECDs that are not suitable for conventional EK surgeries (Table 1). We subsequently demonstrated that the isolated CECs from these donor corneas could be sufficiently pooled for a single application of CE-CI. Through cell injection, we then demonstrated the functionality of these CECs, harvested by means of our SNEC approach, using a previously established rabbit model of bullous keratopathy [37].

In the initial phase of this study, we first optimized the cell-harvesting approach to obtain viable single cells directly from the donor DM. Digestion of the DM (collagenase/liberase) or direct isolation of CECs off the DM using different dissociative enzymatic solutions (dispase, papain, and trypsin alone or in various combinations) generally resulted in (1) the isolation of whole cell sheet or cellular clusters of various sizes, (2) ineffective cellular dissociation, or (3) partial isolation of single cells (Supplementary Figure S1). Even when harsher approaches (such as incubation in an EDTA-buffered solution followed by physical coaxing through a flame-polished pipette to detach the CECs from the DM [56]) were used, cellular viability following isolation and overall cell yield were found to be poor (Supplementary Figure S1).

Eventually, we were able to consistently harvest viable single cells. Cell culture studies have indicated that preincubation of DM/CE in stabilization media improves the success of cultivation of CECs to higher passages and the rates of cellular proliferation [59]. Here, we introduced a 48-h incubation period of donor corneas in a stabilization media (either M4-F99 or M5-Endo), followed by the dissociation of the DM/CE using a mild enzymatic solution (TS) (Figure 1A). Mechanical cell-straining was then performed to eliminate any cellular clusters and potential debris generated from the dissociation. Injecting single cells is crucial, as the injection of cellular clusters or clumps through a 30-gauge needle during cell-injection procedures subjects the CECs to shear stress, which can affect cellular viability [37]. Interestingly, differences were detected when paired donor DM/CEs were stabilized in either of the two media: M4-F99 or M5-Endo (Figure 1B, 1C; Table 2). Whilst single cells could be dissociated from DM/CE incubated in M4-F99 medium in a shorter time (Table 2), the morphological outcomes of the established monolayer were consistently less homogeneous and larger compared to CECs isolated from donor-matched corneas incubated in M5-Endo medium (Figure 1B). Furthermore, quantification of overall cellular yield at week 4 showed that more CECs were obtained following incubation in M5-Endo medium in 2 out of the 3 donor-matched samples (Table 2). As we have shown in previous reports, a homogeneous population of propagated CECs is important in the functional outcomes of cell-based therapies [37]. With the observation that the incubation of isolated DM/CE in M5-Endo medium resulted in CECs with better morphologies and marginally better yield, M5-Endo medium was hence selected as the incubation medium of choice.

Based on the formula to calculate the surface area of a spherical cap: A = 2 π r h, where r = radius of curvature and h = height of cap [60], and working on the assumption that a regular donor cornea has a mean curvature radius of 7.5 mm, with an anterior chamber depth (height) of 3.2 mm, approximately 3.0 × 10^5^ CECs can potentially be isolated from a donor cornea with an ECD of 2000 cells/mm^2^. However, initial characterization studies showed that approximately 65,500, 49,168, and 32,500 CECs could be isolated from donor cornea with ECD of 2619, 2576, and 1454 cells/mm^2^, respectively (Table 3). Factoring in an estimated attrition of 25%, this signified an isolation yield of between 16.7% to 22.1% per donor cornea (Table 3). However, it is important to bear in mind that the estimations of donor ECDs are based on specular microscopy imaging. It must also be noted that current techniques using specular microscopy to estimate donor corneal ECDs have their limitations. Given that only a small area of central cornea is imaged, counted, and inferred to get an ECD quantification, ECD values obtained in this manner may be lower than the actual cellular densities of the entire cornea [61]. Likewise, specular microscopy only gives an image quantification of cells, not their physiological capabilities. Despite this, based on these yields, approximately 1.0 to 1.3 × 10^5^ CECs could thus be potentially isolated from a donor pair with ECD of 2000 cells/mm^2^ for SNEC-injection therapy. This is less than a quarter of the quantity of CECs used in regular CE-CI using CECs propagated in culture, where, in previous studies, at least 5.0 × 10^5^ CECs were injected [37,38,39].

In order to test our hypothesis, we used research-grade donor corneas, where single-cell populations were harvested and used for the in vivo animal experiments; some donor corneas had similarly low ECD (<2500 cells/mm^2^) that are unacceptable for conventional EK procedures (Table 1). Despite the unfavorable donor status, we observed comparable in vivo functional outcomes in our rabbit model of BK receiving regular CE-CI and SNEC injections (Figure 2A). Within the SNEC-injection group, there were also no observable differences in the post-injection ECD (range 923 to 1158 cells/mm^2^) and corneal thicknesses between rabbits’ corneas that received cells harvested from donors <2500 cells/mm^2^, compared to those that received cells harvested from donors with higher ECD. These results indicated that a much lower quantity of CECs from “SNEC” preparation was sufficient to restore corneal endothelial function. Furthermore, there were no detectable differences in time intervals from death of donors to harvesting CECs for SNEC injection, which ranged from 5 to 13 days. Of note, in the current practice of EK procedures, donor corneas preserved in Optisol-GS have a maximum shelf life of 10 days. In our SNEC-injections, three out of 4 of our donors used had shelf lives of ≥10 days.

At week 3, rabbit corneas receiving SNEC injection recovered to mean CCT of 385.5 ± 38.6 μm. This was similar to preoperative levels. Interestingly, CCT in the SNEC-injection group were lower than rabbits that received regular cultured CE-CI, where the corresponding mean CCT at week 3 was 516.0 ± 98.8 μm (*p* = 0.08). This may not be surprising as CECs used in SNEC injection did not undergo cellular expansion and, thus, may be characteristically more similar to the original CE. Indeed, it has been reported that the subculturing of CECs gradually leads to temporal cellular changes and progressive loss of CE-specific gene expression [62], and this may result in a shift in their functional capacities.

From a clinical translational standpoint, a lower number of harvested cells may be sufficient for SNEC injection. In our study, as rabbit CECs are known to be able to regenerate within the eye [63], all native rabbit CECs had to be removed by a complete 12 mm (white-to-white) scraping of rabbits’ DM. This is to prevent rabbit CEC repopulation during the functional assessment of injected human CECs. However, in clinical conditions such as pseudophakic BK or failed corneal grafts, based on the current techniques for treating corneal endothelial failure, only the removal of diseased endothelial cells from the central 7.5 to 8 mm cornea is necessary. Therefore, by targeting cell-injection therapy to the central diseased area of the cornea, the overall quantity of cells required to restore corneal functionality will be significantly lower. To illustrate this, we take an example of donor 15 (Table 1) with low ECD unsuitable for conventional EK surgeries (OD 2342 cells/mm^2^; OS 2273 cells/mm^2^). In our experiment, CECs harvested from donor 15 by an SNEC approach were pooled for cell injection and functionality was demonstrated by a clear rabbit’s cornea at week 3. With an average ECD of 2308 cells/mm^2^ for the paired corneas from donor 15 and assuming a high harvest yield of 22.1%, approximately 76,877 cells per donor (1.54 × 10^5^ cells for a pooled donor pair) could potentially be harvested; we showed that the CECs harvested were able to reverse the effect of corneal blindness even with the entire 12-mm posterior corneal area of native rabbit CECs removed [37]. As the mean post-SNEC-injection ECDs of corneas was approximately 950 cells/mm^2^, this implied that the number of injected cells covering the 12-mm posterior corneal surface was approximately 1.15 × 10^5^. This indicated a cell loss of 25.3% following cell injection. Such loss may be the result of cellular outflow from the eye or nonadherence. Extrapolating from these estimates, approximately 71,800 to 82,000 CECs will thus be required to cover a 7- to 8-mm diseased central area to achieve functionality. It also suggests that donor cornea pairs with an average ECD as low as 1250 cells/mm^2^ could potentially be utilized for SNEC-injection therapy. Future work to validate this should be conducted in animal models of BK with native endothelium that is nonregenerative, such as nonhuman primate [39] or feline [32] models similar to that of human. Also, studies only investigating donor corneas with ECD < 2500 cells/mm^2^ or evaluating CECs harvested from donors stored in other preservation media, such as organ culture media where shelf lives can be as long as 30 days, are required.

The described “SNEC” harvesting technique is a simple process that requires minimal procedural steps and uses reagents that have been approved for use in a first-in-man cell-based therapy clinical trial (www.clinicaltrials.gov NCT04319848) [42]. This can potentially simplify the strict regulatory standards required for the use of in vitro propagated CECs in human clinical trials, where there is a need for more extensive cellular manipulation and where all processes must be performed within a laboratory compliant with Good Manufacturing Practices (GMP) [42]. As the SNEC-injection approach requires minimal cellular manipulation and does not involve cellular propagation, regulatory requirements may be less stringent. With appropriate training, the “SNEC” harvesting processes could be incorporated into standard practice in eye banks that are already routinely preparing corneal graft tissues for EK surgeries (e.g., pre-stripped DMEK). If our harvesting technique is adopted in eye banks worldwide, CECs can be harvested from the pool of donor corneas with low ECDs that are not suitable for conventional EK procedures to treat endothelial diseases. This reduces wastage of donor tissues and the unnecessary cost of organ retrieval.

As mentioned, another pool of corneas that can be used for SNEC harvesting are corneas that have been procured for DMEK procedures. As the DM is highly fragile, harvesting DMEK grafts without damage and wastage of donor tissue poses significant challenges. Studies have reported that certain donor characteristics, such as young donor age [47,48,49], diabetes mellitus [50,51], or previous DM scars [52], are associated with DMEK graft preparation failure due to increased risks of tears in donors’ DM. As a consequence, despite having sufficiently high ECDs (>2500 cells/mm^2^), many corneas harvested from these donors with such unfavorable characteristics are often excluded by eye banks for use in DMEK to avoid unnecessary tissue wastage. For example, 6 out of 22 donors (27.2%) suffered from diabetes mellitus in our study (Table 1) and corneas from these donors would normally be excluded for DMEK in clinical practice. These corneas, however, could be used for SNEC harvesting, as tears in donors’ DM do not exclude the use of these tissues for cell harvesting.

Based on the rapid advancement of research in cell-based therapies for the treatment of corneal endothelial dysfunction, the translation of these therapies into clinical practice as an alternative to conventional corneal transplantation is fast approaching. The scalability of human CECs through cell expansion from a single donor can yield sufficient CECs for the treatment of multiple patients [36,64]. As such, with less reliance on donor availability, cell-based approaches which use cell cultivation modalities present as attractive treatment options to address the global shortage of suitable donor corneas [36,37,42,64]. This alternative technique of “SNEC” harvesting introduced in our study offers the additional advantages (1) of utilizing and repurposing the pool of donor corneas unsuitable for conventional EK surgeries; (2) of a simplified technique of obtaining CECs with minimal manipulation, which can potentially bypass stringent regulatory requirements and the use of specialized GMP facilities; and (3) of being an easier technique of delivering CECs to diseased corneas compared to technically demanding keratoplasty procedures, e.g., DMEK.

## Figures and Tables

**Table 1:** Information of donor corneas procured for this study.

Serial Number	Age	Sex	Days to Process	Cell Count (OS/OD)	Cause of Death	Medical History	Donor Utilization
01 *	59	F	12	2576/N.A.	Cardiopulmonary Arrest	HTN, Throat cancer, anxiety	*Opt (C)*
02 *	34	F	8	1454/N.A.	Anoxia from Drug Overdose	N.A.	*Opt (C)*
03 *	52	F	18	2809/N.A.	Sudden Cardiac Event Congestive Heart Failure	CHF, CKF, HTN, IDDM (w/neuropathy), ischemic cardiomyopathy, hypothyroidism, depression, tobacco use	*Opt (A)*
04 *	71	M	10	N.A./2092	Exsanguination	BCC, HTN, IDDM, chronic tracheostomy, chronic aspiration, hernia with bleeding, esophageal stenosis, hypothyroidism, hyperlipidemia, GI bleed	*Opt (A)*
05 *	69	F	10	2320/N.A.	Acute Cardiogenic Shock	AKI, CAD, CHF, ECMO, HTN, IDDM, MI (x4), PE, LVAD, replacement LVAD, arteriosclerosis, ischemic cardiomyopathy, aortic regurgitation, neuropathy, pulmonary HTN	*Opt (A)*
06 *	66	F	12	2160/N.A.	Cardiac Arrest: Congestive Heart Failure	CHF, COPD, HTN, IDDM, tobacco use	*Opt (A)*
07	70	M	8	1215/2039	Lung Cancer	N.A.	*Opt (A)*
08	65	M	14	2723/3125	Cardiopulmonary Arrest	CAD, CKD, DVT, HTN, chronic back pain, diabetes mellitus, cardiac stents, obesity, nephrectomy	*Opt (A)*
09	28	M	7	2882/2740	Subarachnoid Haemorrhage	Depression, ETOH/tobacco use	*Opt (B)*
10	42	F	12	2447/3454	Multisystemic Failure	Arrythmia, ETOH/tobacco use	*Opt (C)*
11	58	F	17	2670/2568	Acute Cardiac Event	Tobacco use	*Opt (C)*
12	52	F	13	2182/2836	Abdominal/Thoracic Aortic Aneurysm	HTN, Acute Type 1 Aortic dissection, Discoid Lupus, Systemic Lupus, Secondary Raynauds, Acute ischemic stroke	*SNECi*
13	56	F	5	2538/2770	Bronchitis	AKI, CHF, CKD, COPD, HTN, anemia, chronic lymph-edema, restrictive lung disease	*SNECi*
14	54	F	10	2793/2849	Anoxic Brain Injury	Asthma, COPD, ETOH/tobacco use	*SNECi*
15	59	M	11	2342/2273	Intracranial Bleeding/Intracerebral Haemorrhage	HTN, AFib, pericardiac tamponade, hyperlipidemia, RA, GERD, GI bleed, osteoarthritis, tremor disorder, bipolar, depression, ETOH/tobacco use	*SNECi*
16	39	F	14	2833/2857	Central Nervous System Tumour	Brain tumour, seizures, anxiety, ETOH/tobacco use	*Opt (B)*
17	57	F	17	2551/2404	End-stage Renal Disease	AFib, RVR, CAD, CHF, COPD, ESRD, HTN, MI, NIDDM, RLS, hyperlipidemia, polycystic kidney disease, brain aneurysm, GI bleed, skin cancer, anxiety, tobacco use	*Opt (B)*
18	29	F	21	3046/2858	Hanging	N.A.	*Opt (B)*
19	9	M	11	3096/3247	Anoxia	N.A.	*CE-CI*
20	11	F	10	3040/2907	Drowning	N.A.	*CE-CI*
21	23	F	8	2601/2398	Multi-Vehicle Accident	ETOH	*CE-CI*
22	4	F	8	2717/3623	Anoxic Encephalopathy	N.A.	*CE-CI*

AFib: Atrial Fibrillation; AKI: Acute Kidney Injury; BCC: Basal-cell carcinoma; CAD: Coronary Artery Disease; CHF: Congestive Heart Failure; CKF: Chronic Kidney Failure; CKD: Chronic Kidney Disease; COPD: Chronic Obstructive Pulmonary Disease; DVT: Deep vein thrombosis; ECMO: Extracorporeal Membrane Oxygenation; ESRD: End-stage Renal Disease; ETOH: Alcohol use; GERD: Gastroesophageal Reflux Disease; GI: Gastrointestinal; HTN: Hypertension; IDDM: Insulin-dependent Diabetes Mellitus; LVAD: Left Ventricular Assist Device; NIDDM: Non-insulin-dependent Diabetes Mellitus; MI: Myocardial Infarction; OD: Ocular Dextrus (right eye); OS: Ocular Sinister (left eye); RA: Rheumatoid Arthritis; RLS: Restless Leg Syndrome; RVR: Rapid Ventricular Rate.

**Table 2:** Donor-matched DM/CE incubation in either M4-F99 or M5-Endo medium: in vitro culture at week 4.

	Donor 18		Donor 17		Donor 09	
	OS (M4)	OD (M5)	OS (M4)	OD (M5)	OS (M4)	OD (M5)
ECD (cells/mm^2^)	3046	2858	2404	2551	2740	2882
TS Enzymatic Treatment (mins)	35	40	40	50	40	70
Cell Size (μm^2^) ± S.D.	2546.46 ± 1640.65	862.99 ± 462.77	1591.20 ± 773.17	1499.76 ± 681.42	877.58 ± 552.08	632.24 ± 270.23
Cell Circularity Index ± S.D.	0.79 ± 0.09	0.86 ± 0.06	0.85 ± 0.08	0.86 ± 0.07	0.83 ± 0.08	0.86 ±0.06
Overall CEC Count (Week 4)	34,382	91,156	56,662	58,584	95,115	126,985
Overall CEC Yield (Week 4)	1.83 × M5 > M4		0.03 × M4 > M5		0.27 × M5 > M4	

DM: Descemet’s membrane; CE: Corneal endothelium; M4: M4-F99; M5: M5-Endo medium; ECD: Endothelial cell density; TS: Tryple^TM^ Select; CEC: Corneal endothelial cells.

**Table 3:** Percentage yield of isolated single cells from DM/CE incubated in M5-Endo medium.

	Donor 11		Donor 01	Donor 02
	OS	OD	OS	OS
ECD (cells/mm^2^)	2670	2568	2576	1454
Average CEC Density (cells/mm^2^)	2619		N/A	N/A
Theoretical Maximum CECs * (*A* = *2 π r h*)	394,736		388,255	219,147
TS Enzymatic Treatment (mins)	40		35	50
Final Cell Yield (per donor)	65,500		49,168	32,500
Arbitrary Yield (with 25% attrition)	22.1%		16.7%	19.8%

* Based on the formula to calculate the surface area of a spherical cap: A = 2 π r h, where r = radius of curvature and h = height of cap, and working on the assumption that a regular donor cornea has a mean curvature radius of 7.5mm, with an anterior chamber depth (height) of 3.2mm; DM: Descemet’s membrane; CE: Corneal endothelium; M5: M5-Endo medium; ECD: Endothelial cell density; CEC: Corneal endothelial cells; TS: Tryple^TM^ Select.

## Supplementary Materials

The following are available online at https://www.mdpi.com/2073-4409/9/6/1428/s1. Figure S1: Images obtained during optimization phase in unsuccessful trial attempts to directly dissociate the corneal endothelium into single cells using various reagents.
