# Supplementary figures and images for: A Novel Approach of Harvesting Viable Single Cells from Donor Corneal Endothelium for Cell-Injection Therapy
Source: Cells. 2020 Jun 9;9(6):1428. doi: 10.3390/cells9061428 (PMC7349718; doi:10.3390/cells9061428)

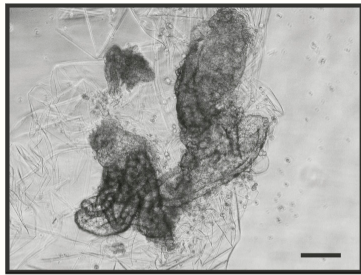

**Collagenase**

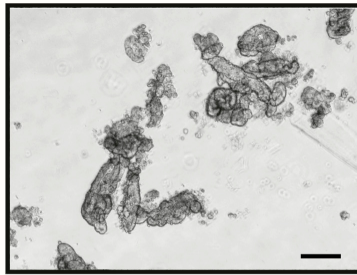

**Papain**

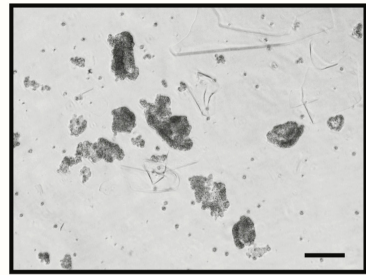

**Dispase**

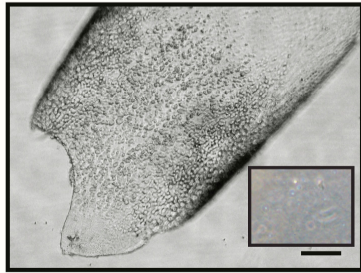

**EDTA**

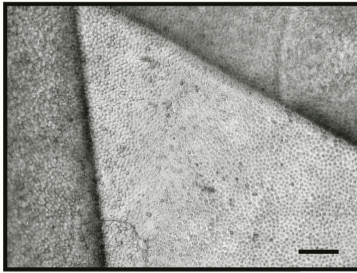

**TrypLE Select**

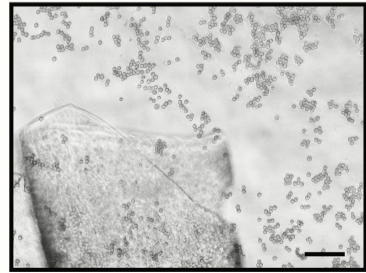

**Papain in TrypLE Select**

Supplement: Supplementary file 1 [file cells-09-01428-s001.pdf]
